# 173 ”You’ve got a lot of big hurdles to jump”: A qualitative investigation into parents’ and teachers’ perceptions of the factors influencing children’s physical activity

**DOI:** 10.1093/eurpub/ckae114.031

**Published:** 2024-09-26

**Authors:** Sarah Nally, A M Gallagher, M H Murphy, Jo Salmon, Angela Carlin

**Affiliations:** Centre for Exercise Medicine, Physical Activity and Health, Sports and Exercise Sciences Research, Institute, Ulster University, Belfast Campus, Belfast, United Kingdom; Nutrition Innovation Centre for Food and Health (NICHE), Biomedical Sciences Research Institute, Ulster University, Coleraine Campus, Coleraine, United Kingdom; Centre for Exercise Medicine, Physical Activity and Health, Sports and Exercise Sciences Research, Institute, Ulster University, Belfast Campus, Belfast, United Kingdom; Physical Activity for Health Research Centre (PHARC), Institute for Sport, Physical Education and Health Sciences, University of Edinburgh, Edinburgh, United Kingdom; School of Exercise and Nutrition Sciences, Deakin University Institute for Physical Activity and Nutrition, Geelong, Australia; Centre for Exercise Medicine, Physical Activity and Health, Sports and Exercise Sciences Research Institute, Ulster University, Magee Campus, Derry, United Kingdom

## Abstract

**Purpose:**

Levels of physical inactivity in children remains a serious public health concern. The school and home setting provide a promising environment to support children’s physical activity (PA). Understanding the factors that influence PA in childhood is key in the development of effective strategies for increasing activity levels and overcoming barriers in primary school aged children. Nevertheless, there is limited evidence in relation to parents’ and teachers’ perceptions of children’s PA behaviour both at school and in the home environment. Thus, the present study aims to explore the current views, barriers, and facilitators to PA in parents and teachers of children (aged 7-9 years) to inform the design of the Children - Sit Less, Move More (C-SLAMM) intervention.

**Methods:**

In total, twelve semi-structured telephone interviews were conducted with primary schoolteachers (n = 6) and with the parents (n = 6) of primary school aged children. All data were audio-recorded, transcribed verbatim and analysed using thematic analysis.

**Results:**

Analyses revealed an understanding of the relationship between children’s PA and health, however the knowledge and perceptions of PA varied. Participants’ felt PA was important but believed several factors impacted children’s ability to be active. Three main themes emerged from the study, reflecting parents’ and teachers’ perspectives on promoting primary school children’s PA at school and at home: (1) knowledge and perception of PA, (2) barriers and facilitators to an active lifestyle, and (3) parent and teacher acceptability and feasibility of the proposed intervention. Findings suggest that the most significant barriers to promoting PA among primary school children include a lack of space, uneven distribution of PA equipment and limited access to play and sporting facilities and suggest opportunities for overcoming these barriers as a way of increasing children’s PA at school. Lack of teacher and parental support were highlighted as key barriers for intervention delivery. Implications for intervention development and future directions were also considered.

**Conclusions:**

Findings from this work highlight that a one-size-fits-all approach may not be applicable when designing a school-based PA intervention, as some degree of flexibility is required.

**Funding Source:**

Northern Ireland Chest Heart and Stroke

